# Investigation of Chemokine Receptor CCR2V64Il Gene Polymorphism and Migraine without Aura in the Iranian Population

**DOI:** 10.1155/2013/836309

**Published:** 2013-12-25

**Authors:** Alireza Zandifar, Maryam Taheriun, Samira Soleimani, Faraidoon Haghdoost, Mohamadhasan Tajaddini, Shaghayegh Haghjooy Javanmard

**Affiliations:** ^1^Medical Student Research Center, Isfahan University of Medical Sciences, Isfahan 81745-319, Iran; ^2^Physiology Research Center, Department of Physiology, Isfahan University of Medical Sciences, Isfahan 81745-319, Iran; ^3^School of Pharmacy and Isfahan Pharmaceutical Sciences Research Center, Isfahan University of Medical Sciences, Isfahan 81745-319, Iran

## Abstract

*Background and Objectives*. Migraine is a multifactorial common neurovascular disease with a polygenic inheritance. Inflammation plays an important part in migraine pathophysiology. C-C chemokine receptor 2 (CCR2) is an important chemokine for monocyte aggregation and transendothelial monocyte migration. The aim of our study was to investigate the association of migraine with CCR2V64Il polymorphism in the Iranian population. *Methods*. We assessed 103 patients with newly diagnosed migraine and 100 healthy subjects. Genomic DNA samples were extracted from peripheral blood and genotypes of CCR2V64Il gene polymorphism were determined. For measuring the severity of headache, every patient filled out the MIGSEV questionnaire. *Results*. There were no significant differences in the distribution of both 64Il allele and heterozygote (GA) genotype of CCR2 gene polymorphism (*P* = 0.396; OR = 0.92, 95% CI = 0.50–1.67 and *P* = 0.388; OR = 0.91, 95% CI = 0.47–1.73, resp.) between case and control groups. There was no significant difference of alleles frequency between three grades of MIGSEV (*P* = 0.922). *Conclusions*. In conclusion our results revealed no association between CCR2V64Il polymorphism and susceptibility to migraine and also headache severity in the Iranian population.

## 1. Introduction

Migraine is a common neurovascular disease characterized by recurrent pulsating and unilateral headache attacks, associated with autonomic symptom such as nausea, vomiting, phonophobia, and photophobia. Some patients have transient neurologic attacks known as “aura” [1]. The prevalence of migraine is about 10% in the general population [2] and the disability of migraine is the same as it is in diabetes mellitus, depression, and myocardial infarction [3].

Migraine is a multifactorial disorder with a polygenic inheritance. Different mechanisms can be related to migraine such as altered sensory input, increased sensitivity of cortex that induces aura, neurogenic inflammation, and hypersensitivity of nociceptors, and sensory neurons that response to pain, in the brain stem [1].

Neurogenic inflammation implicates activation of inflammatory agents and sensitization of nociceptor that innervate the meninge [4].

Chemokines and chemokine receptors play a crucial role in inflammatory processes. Chemokines are proinflammatory cytokines that attract leukocytes and promote accumulation of these cells at the source of chemokine production [5]. Chemokines regulate the migration of monocytes and immature dendritic cells, which express chemokine receptors such as C-C chemokine receptor 2 (CCR2). CCR2 is a member of the CC family that consists of 374 aminoacids and is expressed on endothelial cells, vascular smooth muscles, and macrophages. MCP1 which is a ligand of CCR2, also known as CC Ligand 2 (CCL2), is an important chemokine for aggregation and transendothelial migration of monocytes and also plays an important role in inflammatory diseases and chronic pain [5–7].

CCR2 gene is localized on the chemokine receptor gene cluster on chromosome 3. CCR2A and CCR2B are two alternatively spliced transcript variants. This gene has three exons and spans approximately 8 kb. Most of the studies focus on the role of CCR2 in inflammatory and autoimmune diseases. CCR2 plays a major role in vascular inflammation and developing atherosclerotic plaques [6, 8] and also can modulate risk of arthritis in patient with psoriasis [9].

In recent years most of the studies have focused on the Val64Ile variant of CCR2 [8, 10]. CCR2-V64Il (also known as CCR2 G190A) has a substitution of G to A at base 190 and causes a valine to isoleucine change in position 64 of the first transmembrane domain of the protein [11, 12].

Different studies have investigated the association of CCR2V64Il with several inflammatory diseases and have reported controversial results. CCR2V64Il is associated with coronary artery disease (CAD) [13] and also chronic renal failure (CRF) [11]; on the other hand some other studies show no association between CCR2V64Il and myasthenia gravis, Parkinson's and Alzheimer diseases [12, 14]. There is only one study about migraine and CCR2V64Il which revealed an association between migraine and CCR2 but the association did not remain after adjustment for multiple testing [15]. The aim of our study was to investigate the association of migraine without aura with CCR2V64Il polymorphism in the Iranian population.

## 2. Methods and Material

### 2.1. Patients and Settings

We conducted a case-control study enrolling patients that were recently diagnosed with migraine without aura based on the International Headache Society (IHS) criteria [16] and also controls that were matched for age, education, sex, and socioeconomic status with the cases. The patients were selected from three outpatient neurology clinics between November 2011 and June 2012 in Isfahan, Iran. The sociodemographic and headache characteristics of all the subjects including age, sex, level of education (with and without academic degrees), residency (urban/rural), frequency of headaches, and positive family history were asked. Patients who had at least a three-month history of headaches prior to the diagnoses and who had not have received any medications for their migraine were consecutively enrolled. Controls were selected from healthy people who accompanied the patients that were referred to the neurologic clinics (including patients with migraine and other neurologic disorders) who did not have any history for migraines and also any family history of migraine in the first degree relatives. We did not select any accompanying people who had genetic relation with migraine patients. An informed consent was taken from participants before entering the study. The study was approved by the Ethical Committee of Isfahan University of Medical Sciences.

### 2.2. MIGSEV Questionnaire

Each migraine patient completed a MIGSEV questionnaire as a valid scale for assessing headache severity. The MIGSEV scale, developed by EL Hasnaoui et al. in 2003, is a simple severity scale with four items including intensity of pain, disability in daily activity, tolerability, and nausea that categorizes patients in three groups of intensity: mild, moderate, and severe [17]. This instrument is highly reliable, reproducible, and sensitive that its Persian translation had been used in our previous study as a valid scale [18].

### 2.3. DNA Extraction and Genotyping

Two mL of venous blood was collected from each participant. Genomic DNA samples were extracted from peripheral whole blood using the AccuPrep Genomic DNA Extraction kit (Bioneer Inc., Korea) according to the manufacturer's protocol.

The SNP rs1799864 was identified by the *NCBI* data bank and primers were designed by Beacon Designer 7.91 to flank the coding regions (PREMIER Biosoft International, USA) and synthesized by TIB MOLBIOL, Germany. The forward primer was 5′-ACGGTGCTCCCTGTCATAA-3′ and reverse primer was 5′-CATTCCCAAAGACCCACTCATT-3′.

Genotyping was done by high-resolution melt (HRM) assay using a Rotor-Gene 6000 instrument (Corbett Life Science, Australia).

PCR reactions were carried out in duplicate in 20 *μ*L of final volume using the Type-it HRM kit (Qiagen), HRM PCR buffer, HotStarTaq*Plus* DNA Polymerase, nucleotides and EvaGreen dye, and 30 ng DNA.

The PCR program consisted of an initial denaturation-activation step at 95°C for 5 min, followed by a 40-cycle program (denaturation at 95°C for 15 s, annealing conditions 55°C for 5 seconds, 72°C for 15 seconds; a HRM step from 70 to 95°C rising at 0.1°C per second).

Curves for each duplicate were checked on the shape and peak height to meet reproducibility.

Normalized and temperature-shifted melting curves from HRM, suggestive of single nucleotide polymorphisms (SNP), were distinguished and the samples were subjected to direct sequencing.

### 2.4. Data Analysis

The Hardy-Weinberg equilibrium (HWE) was tested by a goodness-of-fit Chi-square test to compare the observed genotype frequencies to the expected frequencies among controls. We analyzed our data with SPSS software (version 18.0, Chicago, IL). An independent *t*-test was used for quantitative variables between two groups. Relation between polymorphism (homozygous and heterozygous) and different categorized variables, (age, sex, and case-control) was established using Chi-square test and calculation of odds ratio (CI = 95%). The significant level was considered as *P* < 0.05.

## 3. Results

DNA samples from 103 subjects with migraine and 100 healthy subjects were analyzed for CCR2 gene polymorphism. In the case and control groups 82.6% and 78% of participants were female, respectively, and the difference was not statistically significant. Also there were no significant differences in the mean ages (34.08 ± 1.01 versus 34.77 ± 1.06 years), education level (40% versus 36% without academic degrees), and residency (51.5% versus 57.6% urban) between case and control groups, respectively. Subjects' characteristics of the case group are reported in Table 1.

The allele frequencies of G and A in the study population were 357 (87.9%) and 49 (12.1%), respectively. The A allele distribution (Minor Allele Frequency) was not significantly different between migraine patients and control subjects (11.6% versus 12.5%, *P* = 0.396) (Table 2). The frequencies of homozygote and heterozygote genotypes in the study population were 154 (75.9%) and 49 (24.1%), respectively. Frequencies, in migraine and control subjects, were in accord with Hardy-Weinberg equilibrium. There was no significant difference between case and control groups in the frequency of heterozygote genotypes of CCR2 gene (23.3% versus 25%, *P* = 0.388) (Table 2).

The comparison of allele frequencies, between males and females in the case and also control groups, showed that there is no significant difference in the alleles distribution according to the sex (*P* = 0.162 and 0.321, resp.). Further analysis in the migraine subjects showed that the distribution of CCR2 gene polymorphism was not associated with the presence of family history of migraine (*P* = 0.944). We classified all migraine patients into three groups according to the MIGSEV scale. There was no significant difference of alleles frequency between three grades of MIGSEV (*P* = 0.922). Also comparison of the frequency of headache per month between the homozygote and heterozygote patients was not significantly different (8.85 ± 0.96 versus 6.38 ± 1.34, *P* = 0.111).

## 4. Discussion

Results of this study indicated no association between CCR2 polymorphism and migraine; furthermore we found no association between this polymorphism and severity of headache.

Migraine etiology is multifactorial and has polygenic mode of inheritance but neuroinflammation plays an important role in pathophysiology of migraine and causes the deleterious effect in tissue damage progression [19, 20]. Chemokines are released locally from peripheral blood cells in the site of the inflammation and have a key role during inflammatory responses [21]. Chemokines are released locally from peripheral blood cell in site of the inflammation and have a key role during inflammatory responses [22]. In Reuter et al.'s study it has been shown that proinflammatory cytokines enhanced upregulation of nitric oxide synthase (NOS) mRNA. Similarly some other surveys reported the role of chemokines and their receptors in modulation of NO production [23]. NO has an important role in pathophysiology of migraine [24].

TNF-*α* is a proinflammatory cytokine that is related with migraine [19, 25]. Also it has been shown that TNF-*α* is a mediator for releasing CCR2 from endothelial cells, smooth muscle cells, and macrophages [7, 26].

Mitogen activated protein kinase (MAPK) which is an important factor in regulating neural plasticity and inflammatory responses is a target of CCR2 signaling. This fact indicates that CCL2-CCR2 may play a role in neuroinflammation and chronic pain [27]. Also Abbadie et al. indicated the role of CCR2 in excitation nociceptor neuron that increases nociceptor behavior against stimulation which implies that CCR2 has an important role in neuropathic pain [22].

Previous studies indicated a relation between CCR2V64Il, a variant of CCR2, and some inflammatory and autoimmune diseases such as coronary artery disease, diabetes mellitus, progression of HIV, chronic renal failure, and trigeminal neuralgia [8, 11, 27, 28]. These studies showed that CCR2 polymorphism plays a role in CAD and myocardial infarction pathogenesis by vessels inflammation and produced atherosclerotic plaque [8, 29]. In contrary to these studies, some lines of evidence reported protective role of CCR2 in some inflammatory conditions. Miyagishi et al. showed relation between CCR2V64Il and multiple sclerosis that causes reduced progression of multiple sclerosis [30]. Also Huerta et al. found that CCR2V64Il did not contribute to the risk of Parkinson's and Alzheimer diseases [14].

There are only two studies about the relation between migraine and chemokine receptor polymorphisms. Combadière et al. evaluated the relation between headache and CX3CR1 polymorphism. This study showed that CX3CR1 polymorphism had no association with migraine but may play a role in recurrent headaches. This study has some limitations such as misclassification in assessing the type of headache because of recall bias related to elderly participants of this study [31].

Another study was done on the relation between migraine and CCR2V64IL. This study evaluated 77 polymorphisms in a large number of patients with migraine and in contrary to our study showed the correlation of CCR2V64Il and migraine but the association did not remain significant after adjustment for multiple testing. However, this study was conducted only on white females more than 45 years old [15].

In conclusion our result revealed no association between CCR2V64Il polymorphism and susceptibility to migraine without aura and also headache severity in the Iranian population. However, we enrolled newly diagnosed patients by neurologist without the history of receiving any specific medications for migraine which resulted in a relatively small sample size and thus a limitation to our study. The small sample size reduced the power of our study. Especially, in analysis of the association between allele frequency and the three different groups of MIGSEV grades. Therefore this is a preliminary conclusion. Further studies with larger sample sizes from a more diverse ethnic population are needed to confirm these findings.

## Figures and Tables

**Table 1:** Demographic and clinical characteristics of patients with migraine.

Characteristic	Mean ± SE or percentage
Frequency of headache per month	8.32 ± 0.81
Family history for migraine	
Positive	77%
Negative	23%
MIGSEV grade	
Grade I	12.8%
Grade II	38.6%
Grade III	48.6%

**Table 2:** Distribution of allele and genotype of CCR2 gene polymorphism in the case and control groups.

	Case	Control	*P* value	OR (95% confidence interval)
Alleles				
G	182 (88.4%)	175 (87.5%)	0.396	0.92 (0.50–1.67)
A	24 (11.6%)	25 (12.5%)		
Genotype				
Homozygote (GG)	79 (76.7%)	75 (75%)	0.388	0.91 (0.47–1.73)
Heterozygote (GA)	24 (23.3%)	25 (25%)		
